# Prognostic Factors of Postoperative Mortality in Patients with Complicated Right Colon Cancer

**DOI:** 10.3390/life15030350

**Published:** 2025-02-24

**Authors:** Raul Mihailov, Corina Dima, Bianca Georgiana Constantin, Florentin Dimofte, Mihaela Craescu, Lavinia Moroianu, Laura Iuliana Candussi, Virginia Lutenco, Oana Mariana Mihailov, Valerii Lutenco

**Affiliations:** 1 “Dunarea de Jos” Faculty of Medicine and Pharmacy, University of Galati, 800008 Galați, Romania; raul.mihailov@ugal.ro (R.M.); bianca.constantin@ugal.ro (B.G.C.); florentin.dimofte@ugal.ro (F.D.); mihaela.craescu@ugal.ro (M.C.); lavinia.moroianu@ugal.ro (L.M.); iuliana.candussi@ugal.ro (L.I.C.); oana.mihailov@ugal.ro (O.M.M.); valerii.lutenco@ugal.ro (V.L.); 2 “Sf. Apostol Andrei” County Emergency Clinical Hospital, 800008 Galați, Romania; 3 “Dunarea de Jos”Faculty of Sciences and Environment, University of Galati, 800008 Galați, Romania; 4 “Sf. Ioan” Emergency Clinical Hospital for Children, 800008 Galați, Romania; virginia.lutenco@gmail.com

**Keywords:** prognostic factors, postoperative mortality, colon cancer

## Abstract

The incidence of right colon cancer presenting in a stage with complications is significant. There are major differences in therapeutic approach between elective colon cancer surgery and emergency surgery. Complications such as hemorrhage, obstruction, and perforation require careful evaluation of prognostic factors, with morbidity and mortality rates being much higher compared to elective colon surgery. We retrospectively analyzed a group of 95 patients admitted in an emergency to the County Emergency Hospital St. Apostol Apostol Andrei Galati with complicated tumors of the right colon—occlusive, perforated, or hemorrhagic. A series of clinical and biological parameters were followed in order to identify the prognostic factors in the occurrence of death. We analyzed the specialized literature, comparing our study with other similar research from the most important databases. The postoperative death rate in patients with complicated right colon cancer was high. Most complications were occlusive, followed by hemorrhagic and perforative.

## 1. Introduction

In 2020, according to GLOBOCAN, colorectal cancer (CRC) was estimated to account for 10% of the global cancer incidence and 9.4% of cancer deaths, second only to lung cancer, which was estimated to account for 18% of deaths [1,2,3]. Based on the projections of an aging population, population growth, and human development, the global number of new colorectal cancer cases is projected to reach 3.2 million in 2040 [4,5]. In the UK, over 30,000 new cases of colorectal cancers are diagnosed annually [6].

Despite the technical improvements made in recent years, there are still a large number of patients presenting very late to the hospital with complicated tumors, sometimes requiring only palliative interventions. The rate of emergency presentations varies between 8% and 34%, most of which are caused by obstructive and perforated tumors [7,8,9,10,11,12,13].

Complicated right colon cancer represents a pathology with a significant morbidity and mortality rate, especially in emergency surgery. “*Emergency colorectal cancer surgery is recognized to be associated with high morbidity and mortality rates*” [14]. This condition, which may be associated with various complications such as obstruction, perforation, or massive bleeding, requires careful evaluation of the factors influencing the postoperative course of patients. Studies have shown that “*despite advances in surgical techniques and perioperative management, postoperative death rates remain high, particularly among patients with multiple comorbidities or advanced disease*” [9,15,16,17,18].

Compared to patients receiving elective surgery, those requiring emergency surgery face higher morbidity and mortality rates. These poorer outcomes are thought to be caused not only by the nature of the emergency, but also due to the fact that patients presenting as emergent often have greater physiologic imbalances, dehydration, electrolyte disturbances, poor nutritional status, and neglected comorbidities [19,20,21,22]

Colon obstruction complicates between 10 and 19% of colon cancer cases. It is a risk factor for a poor prognosis: the immediate postoperative mortality ranges from 15 to 30%, compared with 1 to 5% for elective surgery, and the morbidity rate is twice as high as for elective surgery [23,24,25,26].

Early detection leads to 5-year survival rates of up to 97.4% for early-stage cancer [27].

On the other hand, late detection with extensive metastatic disease may reduce this survival rate to 8.1% at 5 years [28]. Studies show that at the time of diagnosis, 20% of patients are in the metastatic phase, with the rest of the the podium being occupied by the liver, followed by the lung [29,30,31,32].

The prognosis of patients with complicated rectal colon cancer depends on a multitude of factors that influence the final outcome. Among these, “*the patient’s age, the presence of other chronic conditions (such as cardiovascular disease, diabetes mellitus or chronic renal failure*” [33], the patient’s nutritional status before surgery, and the complexity of the complication are key determinants.

Timing of diagnosis also plays an important role and “delays in identifying complications increase the risk of mortality” [34]. Clinical diagnosis in patients with occlusion is marked by the absence of intestinal transit, vomiting, pain, and flatulence, though sometimes the symptomatology is not specific in early presentations; while in patients with tumor perforations, the intensity of the abdominal pain is greater, and their general condition is greatly influenced. The patient’s functional status before surgery is another critical element. “*Preoperative assessments, such as asa* (American Society of Anesthesiologists) *scores*, *are essential to predict the risks of surgery*” [33]. In addition, “*advanced tumors, the presence of metastases, and lymph node infiltration*” are biological elements that worsen prognosis and increase the risk of death [33].

The surgical techniques used and the experience of the surgical team influence both immediate and long-term outcomes. “Surgery performed in specialized centers by multidisciplinary teams can improve patient prognosis by reducing intraoperative and postoperative complications” [34,35]. These prognostic factors should be taken into account in surgical planning and postoperative management to optimize patient survival and quality of life.

## 2. Material and Methods

We retrospectively analyzed a group of 95 patients admitted in an emergency to the County Emergency Hospital St. Apostol Apostol Andrei Galati with complicated tumors of the right colon—occlusive, perforated, or hemorrhagic. A series of clinical and biological parameters were followed in order to identify prognostic factors in the occurrence of death. Patients hospitalized in chronic services with colon cancer, as well as surgical emergencies for other causes, were excluded from the study. The study sample size provided sufficient data for statistical analysis, but may have limited the generalizability of the findings to a larger population. A larger sample could have provided even more representative results.

Statistical analysis was performed with the IBM SPSS Statistics software package (version 23.0). The significance threshold considered for statistical hypotheses was α = 0.05.

We used the Pearson Chi-Square test and the likelihood ratio to see if there was a connection between death and variables such as gender, background, symptoms, laboratory tests, case mix, intra- and postoperative complications, septic status, the invasion of neighboring organs, and metastases.

In order to check whether there is a correlation between the numerical variables, we calculated the Spearman coefficient and the associated probability.

Two independent sample t-tests were performed in order to investigate whether there were statistically significant differences in the means of the Davies scores, Charlson scores, or number of days of hospitalization between the group of the deceased and the group of other patients.

Kaplan–Meier (KM) is a non-parametric method used to estimate the probability of survival over certain points in time. The main variable is the time measured from the patient’s entry into the study until the critical event occurs (which is death). In addition, the survival distributions of two or more groups, defined in terms of a particular factor, can be compared. The area under curve (AUC) or receiver operating characteristic (ROC) are used to evaluate the performance of a binary classification model, i.e., how well the model is able to distinguish between events and non-events (in our case, death). An AUC value of 0.6–0.69 is considered poor, a value of 0.7–0.79 is considered adequate, an AUC score of 0.8–0.89 is strong, and 0.9–1.0 is excellent.

## 3. Result

**Figure 1:**
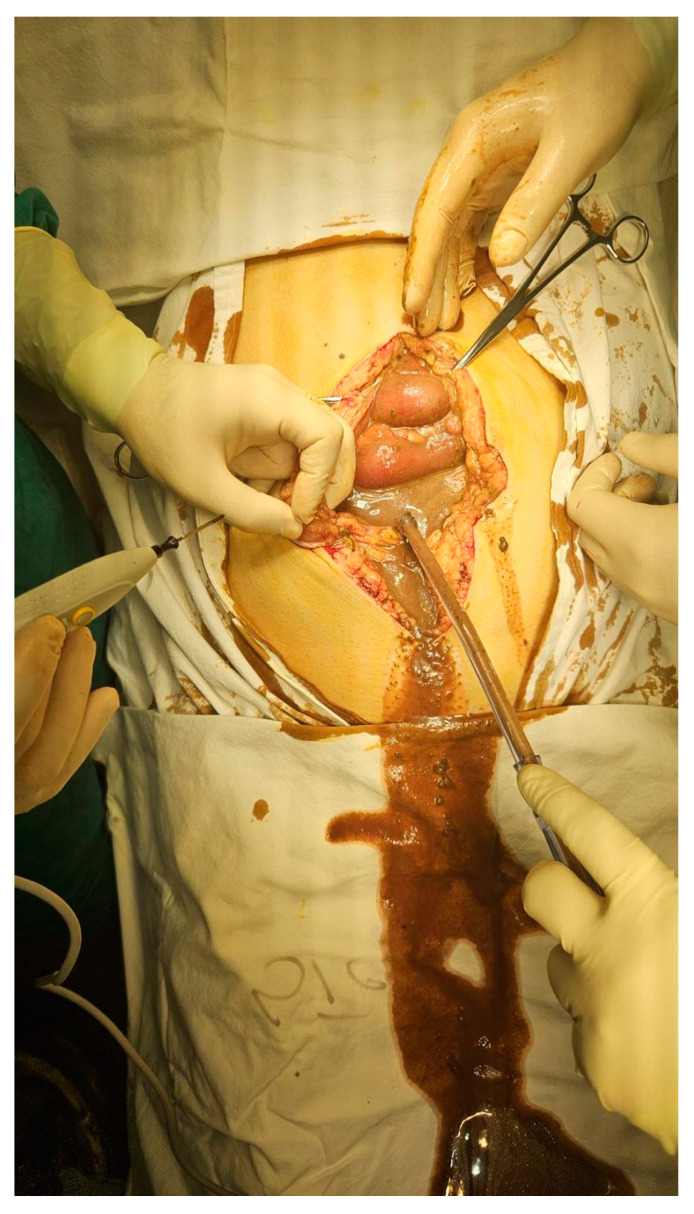
Peritonitis due to the perforation of the right colon cancer (Source: Emergency Clinical Hospital “Sf Apostol Andrei” Galaţi, Surgical Clinic. The patient gave her consent to use her personal data and photos by signing the hospitalization form).

This study analyzes the data obtained from a group of patients aged between 42 and 87 years (mean age = 67.6 years, st. dev. = 10.9218). The group is composed of 45.3% women, 54.7% men, and urban (72.6%) and rural (27.4%) individuals. The symptoms include the following: abdominal pain (94.7%), absence of bowel movements (48.4%), nausea (89.5%), vomiting (64.2%), and weight loss (16.8%). As for laboratory tests, we observe the pathological values of leukocytes (49.5%), anemia (77.9%), the pathological values of platelets (14.7%), the pathological values of blood glucose (27.4%), pathologic creatinine values (40%), electrolyte disturbances (36.8%), acidosis (21.1%), pathologic protein values (18.9%), pathologic albumin values (14.7%), and coagulation disorders (13.7%). Cachexia was present in 15.8% of patients. We encountered OI complications (4.2%), septic state (6.3%), invasion of neighboring organs (15.8%), metastasis (24.2%), and PO complications (27.4%). The postoperative mortality was 12.63% (12/95). (Figure 1)

Table 1 shows patients’ demographics, comorbidities, and laboratory information, and the statistical correlation with postoperative mortality.

The Pearson Chi-Square test was used to determine the degree of association between two categorical variables. Postoperative mortality may be associated with the following: sex (χ^2^ = 7.561, *p* = 0.006), weight loss (χ^2^ = 16.883, *p* < 0.001), type of general condition (χ^2^ = 24.459, *p* < 0.001), cachexia (χ^2^ = 26.739, *p* < 0.001), leukocytes (χ^2^ = 6.299, *p* = 0.012), anemia (χ^2^ = 3.898, *p* = 0.048), platelets (χ^2^ = 20.776, *p* < 0.001), glycemia (χ^2^ = 6.625, *p* = 0.010), creatinine (χ^2^ = 4.070, *p* = 0.044), electrolyte disturbances (χ^2^ = 8.595, *p* = 0.003), total protein (χ^2^ = 4.242, *p* = 0.039), septic status (χ^2^ = 16.944, *p* < 0.001), and PO complications (χ^2^ = 36.450, *p* < 0.001). 

We were unable to establish a relationship between postoperative death and the groups of age (*p* = 0.789), environment of origin (*p* = 0.844), MI.—abdominal pain (*p* = 0.058), MI.—absence of transit (*p* = 0.907), MI.—nausea (*p* = 0.458), MI.—vomiting (*p* = 0.272), HDI (*p* = 0.759), app non neo 1/0 (*p* = 0.160), app neopl 1/0 (*p* = 0.273), onset (*p* = 0.277), acidosis (*p* = 0.061), albumin (*p* = 0.186), coagulation disorder (*p* = 0.222), localization code (*p* = 0.738), DG PREOP (*p* = 0.227), COMPL IO (*p* = 0.447), INT-INTERV (*p* = 0.659), scar abdomen (*p* = 0.162), type of surgery (*p* = 0.353), asoc maneuvers (*p* = 0.112), invasion of neighboring organs (*p* = 0.0449), metastases (*p* = 0.430), preg colon (*p* = 0.058), Reinterv (*p* = 0.503), scheme type (*p* = 0.110), and days of hospitalization (*p* = 0.165).

For the numerical values, we performed the Spearman correlation test and observed that there are correlations between age and number of days of hospitalization (ρ = 0.216, *p* = 0.035), age and Davies score (ρ = 0.294, *p* = 0.004), age and Charlson score (ρ = 0.205, *p* = 0.047), age and adjusted Charlson score (ρ = 0.509, *p* < 0.001), duration of surgery and number of days of hospitalization (ρ = 0.223, *p* = 0.023), number of days of hospitalization and postoperative day of death (ρ = 0.755, *p* = 0.003), Davies score and Charlson score (ρ = 0.654, *p* < 0.001), Davies score and adjusted Charlson score (ρ = 0.550, *p* < 0.001), and Charlson score and adjusted Charlson score (ρ = 0.908, *p* < 0.001) (Table 2).

Table 3 presents an analysis of the mean values of the Davis score, Charlson score, adjusted Charlson score, and number of days of hospitalization for the group of deceased patients compared to the other group of patients.

The *t*-test for the two independent samples shows that there are no statistically significant differences between the mean values of the groups defined by patients who did or did not die postoperatively for the Davies score (t = −1.751, *p* = 0.083, CI = −0.8699–0.0547), for the Charlson score (t = −1.695, *p* = 0.093, CI = −3.2247–0.2549), for the adjusted Charlson score (t = −1.384, *p* = 0.170, CI = −3.3145–0.5916) and for the number of days of hospitalization (t = 1.504, *p* = 0.363, CI = −0.091–0.510). We note, however, that the mean values of the scores are higher for deceased patients and the mean number of days of hospitalization are lower for these patients.

**Figure 2:**
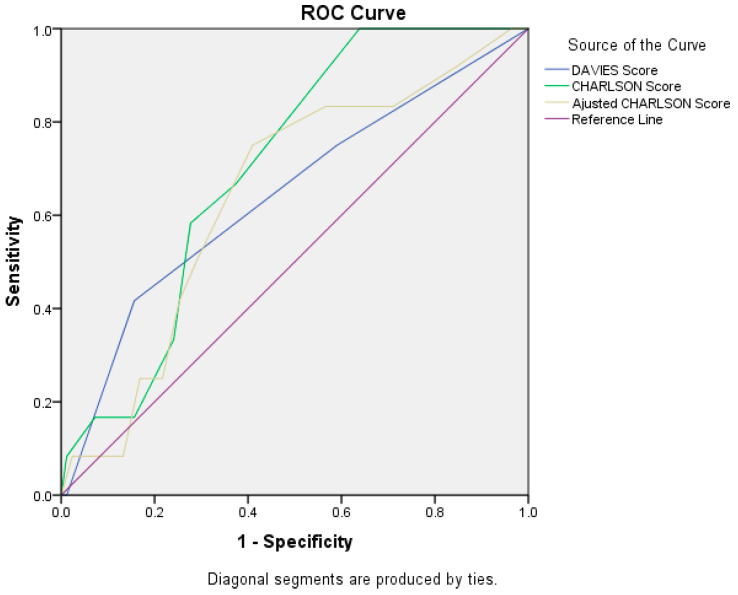
ROC curve for Davies score, Charlson score, and adjusted Charlson score.

The AUC value for the Charlson score was 0.702 (95%CI = 0.579–0.826, *p* = 0.024), which indicates that the Charlson score is an adequate predictor of postoperative mortality. For the Davies score, we obtained AUC = 0.642 (95%CI = 0.464–0.819, *p* = 0.114), and for the Charlson score, we calculated AUC = 0.644 (95%CI = 0.487–0.801, *p* = 0.108) (Figure 2).

**Figure 3:**
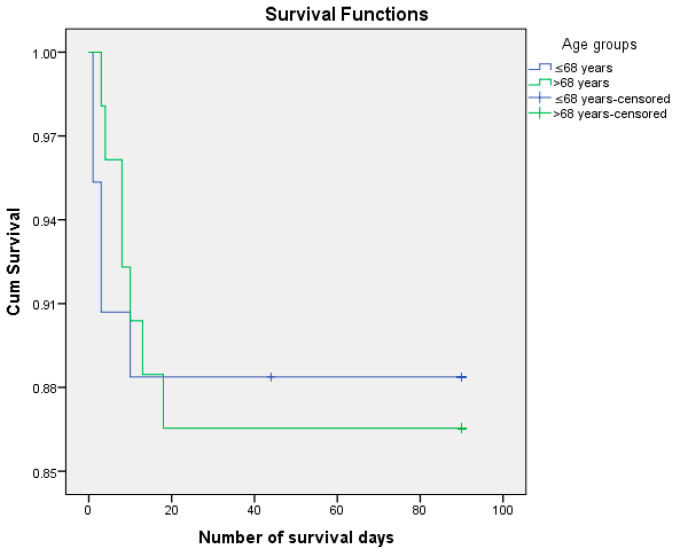
Kaplan–Meier survival curves according to age.

**Figure 4:**
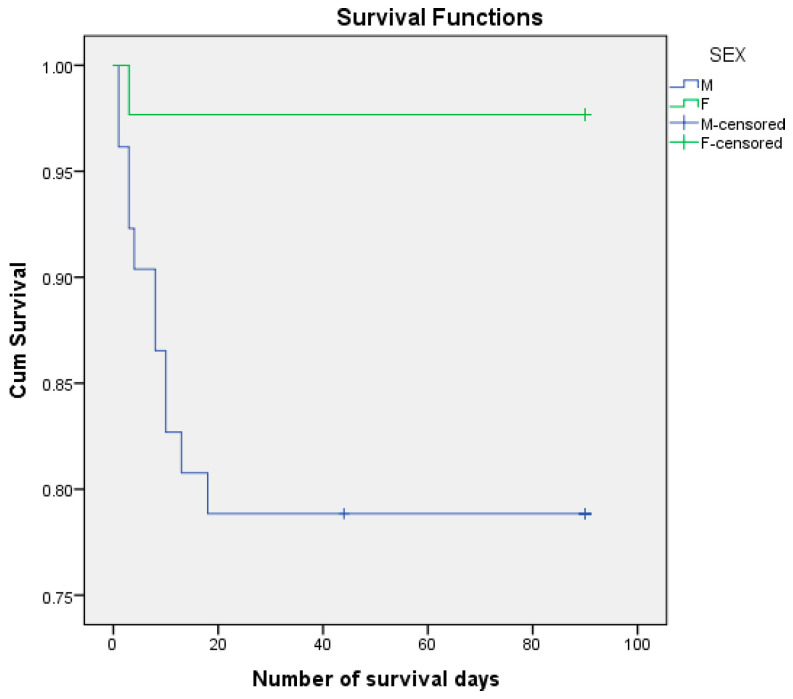
Kaplan–Meier survival curves according to sex.

**Figure 5:**
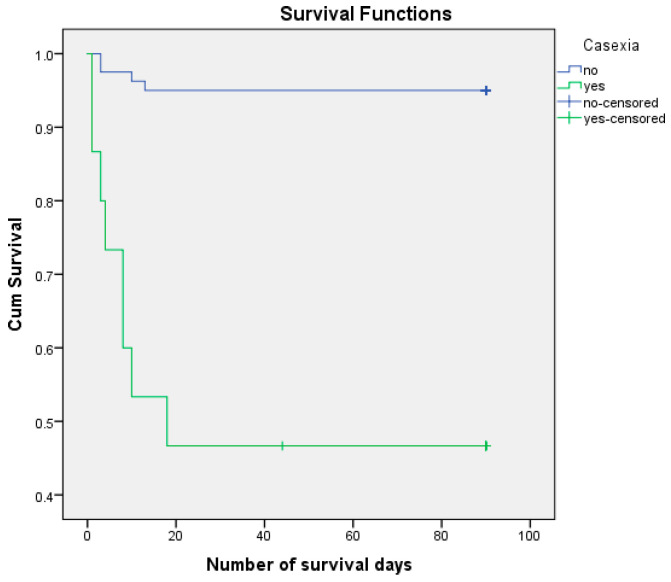
Survival curves Kaplan–Meier according to cachexia.

**Figure 6:**
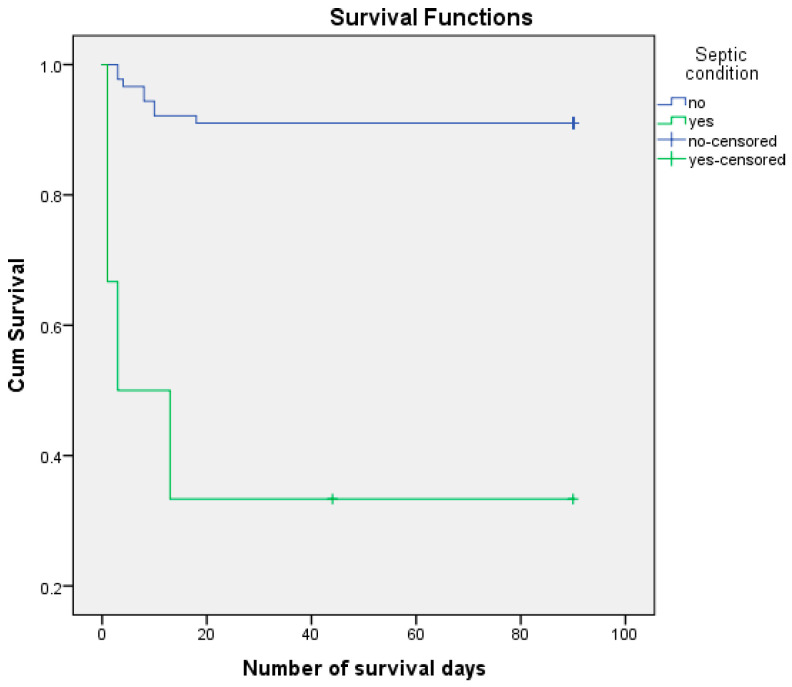
Survival Kaplan–Meier curves according to septic condition.

We analyzed the risk of postoperative death, knowing that 12.6% of patients died postoperatively and 87.4% managed to survive the first 3 months (90 days) after surgery. We performed the survival analysis using the Kaplan–Meier method and the Log Rank (Mantel–Cox) statistical method, considering the number of days of postoperative survival as a time variable. (Figure 3, Figure 4, Figure 5 and Figure 6) We also estimated the mean survival duration. The results obtained were confirmed using Cox Regression (HR). The results are presented in Table 4.

## 4. Discussion

In this study, it was observed that the average age of patients with complicated right colon cancer is 67 years, and they are mainly urban males who present in the emergency room most frequently for abdominal pain, nausea, vomiting, and the absence of bowel transit.

Amri et al. show in a 2015 study show that patients diagnosed and treated in an emergency department for colon cancer present with distinct symptoms from those treated electively. Notably, they present to the emergency room with higher rates of abdominal pain or bloating (56.9% vs. 24.5%) and constipation (13.7% vs. 5.3%). Furthermore, patients who arrive at the emergency room frequently present with severe symptoms, including bowel obstruction (30.4% vs. 2.7%), perforation (15.7% vs. 0.8%), and bleeding (15.7% vs. 1.4%) [36].

Another study conducted in 2021 on a group of 449 patients with complicated colon cancer highlights that the gender distribution of patients shows a predominance of males, with an M/F ratio of 274/175; urban patients are also more numerous, with a U/R ratio of 292/157, data which are comparable to those obtained by us. The distribution by age group reveals that the most affected decades are the eighth and ninth (24% and 36.3%, respectively), and the mean age is 68 years [34,37].

In terms of symptoms, the aforementioned study showed that abdominal pain was present in 96.65% of patients analyzed. Nausea was recorded in 88.41% of patients and the absence of bowel transit in 73.71%. In smaller percentages were vomiting—46.77%, weight loss—22.71%, and only 3.34% of the cases presented with hematochezia.

All patients who underwent stoma or internal shunts had abdominal pain on admission, those with no bowel transit were associated with stoma with or without tumor resection, and those with vomiting had internal shunts. In those with hematochezia, resections with anastomosis were performed. The majority of patients (83.74%) had bowel obstruction; 12.69% had tumor site or diastatic perforations; and 3.56% had hemorrhagic tumors. In patients diagnosed with lower gastrointestinal hemorrhage, resections with anastomosis were performed, those with an obstruction were statistically associated with having an internal bypass, and those with digestive perforations underwent stoma resection.

A small percentage of patients present within the first 24 h of symptom onset. In our study, the percentage was 8.43%; similar studies showed that 6.45% of patients presented to emergency departments in less than 24 h. The aforementioned study also analyzed the correlation between late presentation and surgical intervention, finding that patients with symptom onset within 2–5 days were statistically associated with internal shunts, those with onset between 6 and 14 days had stoma with or without tumor resection, and those with onset more than 14 days later had resection with anastomosis.

The general condition on admission was graded progressively from good to severe according to ECOG status. We found that 21.69% of patients were categorized as ECOG 0, 36.14% ECOG 1, and 32.53% ECOG 2. Another study showed similar results, with only 18.49% patients having had a good general status (ECOG 0), while the others showed various changes: up to 7.35% of patients had a severe general status (ECOG 4). This had an influence on the type of surgery practiced. Thus, in patients with a good or satisfactory general condition, resections with anastomosis were performed; in those with impaired general condition (ECOG2), resections with stoma or internal bypass were performed; and in ECOG3 patients, stomas were performed.

We identified 8.43% of patients with cachexia (estimated by BMI < 18.5). In other studies, the percentage was higher—21.82%, which was statistically associated with ostomies.

In our study, the patients had changes in biological parameters, changes which were found in other studies in which laboratory tests showed changes in leukocytes, and that 65.29% patients had anemia on admission, 14.7% had thrombocytosis/thrombocytopenia, 22.27% had changes in blood glucose on admission, 39.42% had increases in creatinine, 30.06% had electrolyte disturbances, 21.82% had metabolic acidosis, 11.80% of patients had coagulation disorders, and 9.13% had a septic state on admission [34].

We did not observe the statistical significance for survival of the following factors: age group (*p* = 0.845), background (*p* = 0.835), MI.—abdominal pain (*p* = 0.845), MI. (*p* = 0.071), MI.—absence of transit (*p* = 0.897), MI.—nausea (*p* = 0.487), MI.—vomiting (*p* = 0.317), HDI (*p* = 0.769), app non neo 1/0 (*p* = 0.152), app neopl 1/0 (*p* = 0.267), onset (*p* = 0.459), anemia (*p* = 0.054), albumin (*p* = 203), coagulation disorders (*p* = 0.196), localization code (*p* = 0.728), DG PREOP (*p* = 0.109), COMPL IO (*p* = 0.497), INT-INTERV (*p* = 0.708), scar abdomen (*p* = 0.157), type of surgery (*p* = 0.240), asoc manvre (*p* = 0.077), invasion of neighboring organs (*p* = 0.465), metastases (*p* = 0.393), preg colon (*p* = 0.053), reinterv (*p* = 0.517), type of scheme (*p* = 0.602), and days of hospitalization (*p* = 0.147).

Of the female patients, 2.33% died postoperatively, compared to the male patients of whom 21.15% died. The mean estimated number of days of survival was 72,481 days for males and 87,977 days for females. The mortality risk associated with sex was HR = 0.102, 95% CI (0.013, 0.787) (*p* = 0.029).

We observed an increased risk of postoperative death associated with weight loss—HR = 8.941, 95% CI (2.824, 28.303) (*p* < 0.001). A total of 6.32% of patients without weight loss and 43.75% of patients with weight loss died. The mean estimated postoperative survival was 84.899 days for non-weight loss patients and 52.813 days for weight loss patients.

Cachexia may be associated with the risk of postoperative death—HR = 14.179, 95% CI (4.246, 47.346)) (*p* < 0.001). A total of 53.33% of patients with cachexia died postoperatively and only 5.0% of patients without cachexia died postoperatively. The estimated number of days of survival in patients with cachexia was 45,533, and in those without cachexia was 85,863.

The postoperative death rate was 26.92% in patients with glycemia and 7.25% in other patients. The median number of estimated survival days was 67,538 in patients with glycemia and 84,000 in the others. The mortality risk associated with glycemia was HR = 0.243, 95% CI (0.077, 0.766) (*p* = 0.016).

The percentage of postoperative deaths was 21.05% in patients with creatinine and 7.02% in other patients. The median number of days of estimated survival was 72,395 in creatinine patients and 84,228 in the others. The mortality risk associated with creatinine was HR = 0.312, 95% CI (0.094, 1.037) (*p* = 0.047).

The risk of mortality associated with electrolyte disturbances was HR = 5.711, 95% CI (1.545, 21.105) (*p* = 0.009). The median number of estimated survival days was 68,600 for those with electrolyte disturbances and 85,850 for the remaining patients. Percentagewise, 27.71% of patients with electrolyte disturbances and 5.00% of the remaining patients died.

We determined a risk of postoperative death associated with acidosis—HR = 2.972, 95% CI (0.942, 9.371) (*p* = 0.048). The median number of days of survival estimated for patients with acidosis was 68,750 and 82,360 for the remaining patients. Postoperatively, 25.00% of patients with acidosis and 9.33% of the remaining patients died.

The risk of postoperative death associated with sepsis was HR = 12.186, 95% CI (3.631, 40.895) (*p* < 0.001). The median number of days of survival for patients with a septic status was estimated at 33.000 and 82.629 for the remaining patients. Postoperatively, 66.67% of patients with acidosis and 8.98% of the remaining patients died. It should be noted that a septic state was present in only 6.32% of the sample analyzed.

Tumors located on the right side of the colon are reported to be more advanced at diagnosis. In a smaller study, tumor location was not associated with significant differences in survival.

However, in a large prospective cohort study in the USA of nearly 78,000 patients operated on for colon cancer, postoperative mortality was found to be higher in patients with right colon tumors than in those with left colon tumors (4.0% vs. 5.3%). Also, the median age was higher for patients with right colon tumors (73 vs. 69 years), the proportion of poorly differentiated tumors was higher (25% vs. 14%), and the median survival was lower (78 months vs. 89 months). Adjusted multivariate analysis showed that the risk of death within five years was higher for right colon tumors. These data emphasize the need for a differentiated approach in the management of right versus left colon cancer, given the unique characteristics and different impacts on patient survival [38,39].

Another recent study conducted in Romania in 2022 identifies the following causes of death in patients with complicated colon cancer: septic shock, congestive heart failure, myocardial infarction, anastomotic fistula, acute respiratory failure, acute renal failure, and bowel obstruction. Nine independent predictors of 30-day postoperative mortality were identified in a multivariate logistic analysis model: age > 70 years, congestive heart failure, ECOG > 2 (Eastern Cooperative Oncology Group performance status), sepsis, obesity, cachexia, abnormal platelet values, abnormal creatinine values, and proximal tumors [40].

The association of comorbidities with the occurrence of postoperative complications in patients with colon cancer is also supported by the literature data. Among pre-existing conditions, liver cirrhosis, chronic liver disease, obesity, diabetes, a history of operated digestive cancer, other major abdominal surgery, pulmonary disease, and pre-existing renal failure are factors that increase morbidity [14,40,41,42,43].

Another study mentions congestive heart failure and chronic renal failure as being associated with higher death rates in emergency colorectal surgery [44,45,46].

Nilsen et al. showed in a study published in 2021 and conducted on a large number of patients that among patients with colorectal cancer, an older age, advanced disease stage, and a higher level of comorbidities were significantly associated with increased odds of being diagnosed in an emergency presentation. Patients with locally advanced or metastatic colorectal cancer were 2-fold and 5-fold more likely, respectively, to be diagnosed after an emergency presentation compared to patients with localized disease, and patients with colon cancer were 2.7-fold more likely to be diagnosed on emergency presentation compared to patients with rectal cancer. The one-year overall survival rate for patients who had an emergency presentation prior to colorectal cancer diagnosis was 67.7%, whereas for patients without an emergency presentation it was 90.2% [47].

In our study, although the age-adjusted Davies, Charlson, and Charlson comorbidity scores had higher values in the patients who died, we could not assess their statistical significance. Despite our findings, other studies have shown that a significant number of comorbidities is one of the most important predictors of postoperative complications and early mortality [48].

Another study analyzing the prognostic factors in complicated colorectal cancer mentions associated cardiac pathology (especially fibrillation) as one of the most important risk factors [49,50,51].

Analyzing the risks of death and survival of patients with complicated colorectal cancer, Constantin et al. showed that inflammation-based prognostic scores such as NLR (neutrophil/lymphocyte ratio), PLR (platelet/lymphocyte ratio), LMR (lymphocyte/monocyte ratio) PNI (prognostic nutritional index), and SIR scores were associated with the survival of colorectal tumor patients. In univariate analysis, high values of NLR and PLR were found to be risk factors and high values of LMR and PNI were found to be protective factors for the survival of colorectal tumor patients undergoing emergency surgery. An increased PLR value is an independent risk factor for patients in the group, while increased values of LMR and PNI are independent protective factors for survival [52].

In a 2023 study of 391 patients with complicated colorectal cancer, Constantin et al. showed that the NLR inflammation-based prognostic score, an outcome of a systemic inflammatory response, was associated with patient survival. In the univariate analysis, we found that elevated NLR values were risk factors for survival in colorectal cancer patients undergoing emergency surgery [53].

Although no statistically significant correlation between the presence of metastases and the occurrence of postoperative death was obtained in our study group, Honghua Peng et al. find that liver metastases are a poor prognostic factor for survival [54].

Studying the problem of survival in patients with advanced colon cancer, Taeyeong Eom et al. showed through their study a contribution to the understanding of the prognostic factors in T4 colon cancer, emphasizing that en bloc resection, surgical techniques, and adjuvant chemotherapy play a significant role in improving prognosis. Although no significant differences in outcome were observed between T4a and T4b in curative surgery, the type of surgery (laparoscopic vs. open) and the use of adjuvant chemotherapy were important factors in long-term survival [55].

The data obtained in this analysis of the prognostic factors for complicated right colon cancer could be used to reduce postoperative morbidity and mortality, opting to prompt intervention limited to emergency surgical procedures.

In order to obtain the best possible results, we believe that it is appropriate for patients with complicated cancers, with multiple associated pathologies that require a multidisciplinary approach, to be transferred to higher ranking centers [56].

Regarding future research directions, we believe that a similar study targeting complicated left colon cancer would be beneficial in adapting diagnostic and treatment strategies. Also, comparing the results between the two studies would bring benefits in case management. A multicenter study of these topics would also be relevant.

## 5. Conclusions

The postoperative death rate in patients with complicated right colon cancer is high. Most complications were occlusive, followed by hemorrhagic and perforative. Less than 10% of patients presented within the first 24 h of symptom onset. The most relevant prognostic factors in the occurrence of death were weight loss, cachexia, renal failure, electrolyte disturbances, and metabolic acidosis.

## Figures and Tables

**Table 1:** Correlation between postoperative deaths and demographic data and laboratory analyses.

		Postoperative Deaths—No	Postoperative Deaths—Yes	*p*-Value (Test)
Age group	≤68 years	38/83 (45.78%)	5/12 (41.67%)	0.789 ^(1)^
	>68 years	45/83 (54.22%)	7/12 (58.33%)	
Sex	M	41/83 (49.40%)	11/12 (91.67%)	0.006 ^(1)^
	F	42/83 (50.60%)	1/12 (8.33%)	
Urban/rural area	U	60/83 (72.29%)	9/12 (75.00%)	0.844 ^(1)^
	R	23/83 (27.71%)	3/12 (25.00%)	
Abdominal pain	No	3/83 (3.61%)	2/12 (16.67%)	0.058 ^(1)^
	Yes	80/83 (96.39%)	10/12 (83.33%)	
Absence of transit	No	43/83 (51.81%)	6/12 (50.00%)	0.907 ^(1)^
	Yes	40/83 (48.19%)	6/12 (50.00%)	
Nausea	No	8/83 (9.64%)	2/12 (16.67%)	0.458 ^(1)^
	Yes	75/83 (90.36%)	10/12 (83.33%)	
Vomiting	No	28/83 (33.73%)	6/12 (50.00%)	0.272 ^(1)^
	Yes	55/83 (66.27%)	6/12 (50.00%)	
Weight loss	No	74/83 (89.16%)	5/12 (41.67%)	0.000 ^(1)^
	Yes	9/83 (10.84%)	7/12 (58.33%)	
Lower digestive hemorrhage	No	78/83 (93.98%)	11/12 (91.67%)	0.759 ^(1)^
	Yes	5/83 (6.02%)	1/12 (8.33%)	
Personal history of non-neoplastic pathology 1/0	No	31/83 (37.35%)	2/12 (16.67%)	0.160 ^(1)^
	Yes	52/83 (62.65%)	10/12 (83.33%)	
Personal history of neoplastic pathology 1/0	No	81/83 (97.59%)	11/12 (91.67%)	0.273 ^(1)^
	Yes	2/83 (2.41%)	1/12 (8.33%)	
Onset of symptoms	≤1 day	7/83 (8.43%)	0/12 (0.00%)	0.277 ^(2)^
	2–5 days	47/83 (56.63%)	6/12 (50.00%)	
	>14 days	29/83 (34.94%)	6/12 (50.00%)	
Type of general status	0	18/83 (21.69%)	0/12 (0.00%)	0.000 ^(2)^
	1	30/83 (36.14%)	1/12 (8.33%)	
	2	27/83 (32.53%)	4/12 (33.33%)	
	3	6/83 (7.23%)	3/12 (25.00%)	
	4	2/83 (2.41%)	4/12 (33.33%)	
Cachexia	No	76/83 (91.57%)	4/12 (33.33%)	0.000 ^(1)^
	Yes	7/83 (8.43%)	8/12 (66.67%)	
Leukocytes	N	46/83 (55.42%)	2/12 (16.67%)	0.012 ^(1)^
	P	37/83 (44.58%)	10/12 (83.33%)	
Anemia	No	21/83 (25.30%)	0/12 (0.00%)	0.048 ^(1)^
	Yes	62/83 (74.70%)	12/12 (100.00%)	
Platelets	P	7/83 (8.43%)	7/12 (58.33%)	0.000 ^(1)^
	N	76/83 (91.57%)	5/12 (41.67%)	
Blood glucose	P	19/83 (22.89%)	7/12 (58.33%)	0.010 ^(1)^
	N	64/83 (77.11%)	5/12 (41.67%)	
Creatinine	P	30/83 (36.14%)	8/12 (66.67%)	0.044 ^(1)^
	N	53/83 (63.86%)	4/12 (33.33%)	
Electrolyte disorders	No	57/83 (68.67%)	3/12 (25.00%)	0.003 ^(1)^
	Yes	26/83 (31.33%)	9/12 (75.00%)	
Acidosis	No	68/83 (81.93%)	7/12 (58.33%)	0.061 ^(1)^
	Yes	15/83 (18.07%)	5/12 (41.67%)	
Total proteins	N	10/83 (12.05%)	0/12 (0.00%)	0.039 ^(1)^
	P	12/83 (14.46%)	6/12 (50.00%)	
Albumin	N	7/83 (8.43%)	0/12 (0.00%)	0.186 ^(1)^
	P	11/83 (13.25%)	3/12 (25.00%)	
Coagulation disorders	No	73/83 (87.95%)	9/12 (75.00%)	0.222 ^(1)^
	Yes	10/83 (12.05%)	3/12 (25.00%)	
Location code	C18.0	36/83 (43.37%)	6/12 (50.00%)	0.738 ^(2)^
	C18.2	17/83 (20.48%)	3/12 (25.00%)	
	C18.3	30/83 (36.14%)	3/12 (25.00%)	
Preoperative diagnosis	H	8/83 (9.64%)	1/12 (8.33%)	0.227 ^(2)^
	O	69/83 (83.13%)	8/12 (66.67%)	
	P	6/83 (7.23%)	3/12 (25.00%)	
Intraoperative complications	No	80/83 (96.39%)	11/12 (91.67%)	0.447 ^(1)^
	Yes	3/83 (3.61%)	1/12 (8.33%)	
Septic status	No	81/83 (97.59%)	8/12 (66.67%)	0.000 ^(1)^
	Yes	2/83 (2.41%)	4/12 (33.33%)	
Interval-intervention	<12 h	36/83 (43.37%)	6/12 (50.00%)	0.659 ^(2)^
	12–24 h	15/83 (18.07%)	1/12 (8.33%)	
	>24 h	32/83 (38.55%)	5/12 (41.67%)	
Scar abdomen	No	64/83 (77.11%)	7/12 (58.33%)	0.162 ^(1)^
	Yes	19/83 (22.89%)	5/12 (41.67%)	
Operation type	1	2/83 (2.41%)	1/12 (8.33%)	0.353 ^(2)^
	3	17/83 (20.48%)	4/12 (33.33%)	
	4	64/83 (77.11%)	7/12 (58.33%)	
Associated maneuvers	-	71/83 (85.54%)	7/12 (58.33%)	0.112 ^(2)^
	Major	7/83 (8.43%)	3/12 (25.00%)	
	Minor	5/83 (6.02%)	2/12 (16.67%)	
Other organ invasion	No	69/83 (83.13%)	11/12 (91.67%)	0.449 ^(1)^
	Yes	14/83 (16.87%)	1/12 (8.33%)	
Metastases	No	64/83 (77.11%)	8/12 (66.67%)	0.430 ^(1)^
	Yes	19/83 (22.89%)	4/12 (33.33%)	
Preparation of colon	No	3/83 (3.61%)	2/12 (16.67%)	0.058 ^(1)^
	Yes	80/83 (96.39%)	10/12 (83.33%)	
Postoperative complications	No	69/83 (83.13%)	0/12 (0.00%)	0.000 ^(1)^
	Yes	14/83 (16.87%)	12/12 (100.00%)	
Reinterventions	No	80/83 (96.39%)	12/12 (100.00%)	0.503 ^(1)^
	Yes	3/83 (3.61%)	0/12 (0.00%)	
Scheme type	1	44/83 (53.01%)	4/12 (33.33%)	0.110 ^(2)^
	2	23/83 (27.71%)	5/12 (41.67%)	
	3	12/83 (14.46%)	2/12 (16.67%)	
	4	4/83 (4.82%)	1/12 (8.33%)	
Number of hospitalization days	<15 days	31/83 (37.35%)	7/12 (58.33)	0.165 ^(2)^
	≥15 days	52/83 (62.65%)	5/12 (41.67)	
Total		83	12	

^(1)^ Pearson Chi-Square; ^(2)^ likelihood ratio.

**Table 2:** Descriptive analysis of parameters.

Descriptive Statistics					
	Minimum	Maximum	Mean	Std. Deviation	Variance
Age	42.0	87.0	67.600	10.9218	119.285
Duration of surgical intervention (hours)	1.0	4.0	2.205	0.5812	0.338
Davies score	1.0	4.0	1.811	0.7621	0.581
Charlson score	2.0	11.0	4.453	2.8649	8.208
Charlson adjusted score	6.0	18.0	10.811	3.2000	10.240
Number of hospitalization days	1.0	48.0	16.084	7.0328	49.461

**Table 3:** Correlations between patient comorbidities and number of days of hospitalization.

Postoperative Deaths		N	Mean	Std. Deviation	Std. Error Mean
Davies score	NO	83 (87.37%)	1.759	0.7423	0.0815
	YES	12 (12.63%)	2.167	0.8348	0.2410
Charlson score	NO	83 (87.37%)	4.265	2.8331	0.3110
	YES	12 (12.63%)	5.750	2.8644	0.8269
Charlson adjusted score	NO	83 (87.37%)	10.639	3.1990	0.3511
	YES	12 (12.63%)	12.000	3.0748	0.8876
Number of hospitalization days	NO	83 (87.37%)	16.494	6.0975	0.6693
	YES	12 (12.63%)	13.250	11.6395	3.3600

**Table 4:** Survival duration.

		Mean Estimate	*p*-Value Log Rank (Mantel–Cox)	Univariate	
				HR (95%CI)	*p*-Value
Age group	≤68 years	79.953	0.845	1.120 (0.355, 3.529)	0.846
	>68 years	79.115			
Sex	M	72.481	0.007	0.102 (0.013, 0.787)	0.029
	F	87.977			
Urban/rural area	U	79.145	0.835	0.871 (0.236, 3.271)	0.836
	R	80.423			
Abdominal pain	NO	59.200	0.071	0.273 (0.060, 1.246)	0.094
	YES	80.622			
Absence of transit	NO	79.857	0.897	1.077 (0.347, 3.339)	0.898
	YES	79.109			
Nausea	NO	73.800	0.487	0.589 (0.129, 2.690)	0.495
	YES	80.165			
Vomiting	NO	75.912	0.317	0.567 (0.183, 1.758)	0.326
	YES	81.492			
Weight loss	NO	84.899	0.000	8.941 (2.824, 28.303)	0.000
	YES	52.813			
Lower digestive hemorrhage	NO	79.708	0.769	1.355 (0.175, 10.496)	0.771
	YES	76.333			
Personal history of non-neoplastic pathology 1/0	NO	85.242	0.152	2.867 (0.628, 13.087)	0.174
	YES	76.435			
Personal history of neoplastic pathology 1/0	NO	80.043	0.267	2.995 (0.386, 23.239)	0.294
	YES	62.667			
Onset of symptoms	≤1 day		0.459	1.344 (0.790, 2.285)	0.276
	2–5 days				
	>14 days				
Cachexia	NO	85.863	0.000	14.179 (4.246, 47.346)	0.000
	YES	45.533			
White blood cells	N	86.542	0.012	5.565 (1.219, 25.408)	0.027
	P	72.298			
Anemia	NO		0.054	30.467 (0.101, 9195.175)	0.271
	YES				
Platelets	P	48.500	0.000	0.097 (0.031, 0.307)	0.000
	N	84.852			
Blood glucose	P	67.538	0.008	0.243 (0.077, 0.766)	0.016
	N	84.000			
Creatinine	P	72.395	0.043	0.312 (0.094, 1.037)	0.047
	N	84.228			
Electrolyte disorders	NO	85.850	0.003	5711 (1.545, 21.105)	0.009
	YES	68.600			
Acidosis	NO	82.360	0.048	2.972 (0.942, 9.371)	0.048
	YES	68.750			
Coagulation disorders	NO	80.951	0.196	2.299 (0.622, 8.496)	0.212
	YES	70.308			
Location code	C18.0	77.833	0.728	0.873 (0.572, 1.330)	0.526
	C18.2	77.550			
	C18.3	82.788			
Preoperative diagnosis	H	80.889	0.109	2.601 (0.751, 9.002)	0.131
	O	81.390			
	P	61.889			
Intraoperative complications	NO	79.879	0.497	1.988 (0.258, 15.482)	0.508
	YES	70.750			
Septic status	NO	82.629	0.000	12.186 (3.631, 40.895)	0.000
	YES	33.000			
Interval-interventions	<12 h	77.881	0.708	0.944 (0.506, 1.762)	0.857
	12–24 h	84.563			
	>24 h	79.135			
Scar abdomen	NO	81.718	0.167	2.191 (0.695, 6.906)	0.181
	YES	72.917			
Operation type	1	61.000	0.240	0.599 (0.323, 1.110)	0.103
	3	73.714			
	4	81.986			
Associated maneuvers	-	82.436	0.077	0.292 (0.061, 1.407)	0.125
	Major	65.900			
	Minor	66.143			
Other organ invasion	NO	78.613	0.465	0.476 (0.061, 3.690)	0.478
	YES	84.200			
Metastases	NO	80.958	0.393	1.673 (0.504, 5.561)	0.401
	YES	74.913			
Colon preparation	NO	57.200	0.053	0.252 (0.055, 1.150)	0.075
	YES	80.733			
Scheme type	1	83.063	0.602	1.378 (0.776, 2.446)	0.274
	2	75.357			
	3	78.143			
	4	72.200			
Number of hospitalization days	<15 days	74.316	0.147	0.440 (0.140, 1.386)	0.161
	≥15 days	82.947			

## Data Availability

Data are contained within the article.
